# Exploring barriers and facilitators to immediate postpartum intrauterine device uptake within the strengthening Egypt family planning program: a case-control study

**DOI:** 10.1186/s12913-025-13306-3

**Published:** 2025-08-14

**Authors:** Mireille M. Hanna, Omaima El Gibaly, Mohamed M. F. Fathalla, Heba M. Mohammed

**Affiliations:** 1 Public Health and Community Medicine Department, Faculty of Medicine, Assiut University, Assiut City, 71515 Egypt; 2 Obstetrics and Gynecology Department, Faculty of Medicine, Assiut University, Assiut City, 71515 Egypt

**Keywords:** IPPIUD (Immediate postpartum intrauterine device), Barriers, Predictors, Uptake, Counseling, Family planning

## Abstract

**Background:**

Egypt faces significant economic and healthcare challenges due to overpopulation. The Strengthening Egypt Family Planning Program tackles the rapid population growth by improving access to family planning services. Given Egypt’s high cesarean section rates, the program leverages this opportunity to facilitate immediate postpartum IUD (IPPIUD) insertion as a safe and efficient method. This study identifies barriers and predictors of IPPIUD uptake, providing valuable insights to enhance family planning services in Egypt and other low- and middle-income countries facing overpopulation, such as Kenya, India, and Ethiopia, which have implemented similar initiatives.

**Methods:**

A case-control study was conducted on women who underwent cesarean sections in three public hospitals in Assiut City, Egypt. The study included two groups: 210 cases (women who used IPPIUD), and 210 controls (women who did not). Data was collected using a semi-structured questionnaire covering sociodemographic data, obstetric history, reproductive history, and barriers to IPPIUD uptake. Statistical analyses employed Chi-square tests, t-tests, and logistic regression to identify predictors of IPPIUD use.

**Results:**

The study found that the main barriers to IPPIUD use were disapproval from husbands (73.3%), desire for more children (71.9%), lack of knowledge about IPPIUD (67.6%), preference for female providers (64.3%), and negative provider attitudes (20.0%), fear of side effects (27.6%), and misconceptions (22.4%). The significant predictors of IPPIUD use were receiving IPPIUD counseling during antenatal care (AOR = 11.42, 95% CI: 4.58–28.49), having husband’s support for family planning (AOR = 9.43, 95% CI: 3.44–25.83), receiving IPPIUD counseling during labor (AOR = 5.63, 95% CI: 2.56–12.33), and having sons (AOR = 4.24, 95% CI:1.37–13.14), their source of knowledge about FP is from ANC (AOR = 2.39, 95% CI: 1.89–5.83), high socioeconomic status (AOR = 1.54, 95% CI: 1.23–1.95), increased women’s age (AOR = 1.13, 95% CI: 1.06–1.21).

**Conclusions:**

Barriers to IPPIUD use include husbands’ disapproval, desire for more children, lack of knowledge, preference for female providers, provider negativity, and fear of side effects. Key predictors of uptake are counseling during antenatal care and labor, husband’s support, and having sons. Policymakers should enhance family planning education targeting men and address gender norms, while providers must leverage every opportunity during antenatal, labor, and postpartum care to provide counseling.

**Trial registration:**

Number: NCT05471362.

**Supplementary Information:**

The online version contains supplementary material available at 10.1186/s12913-025-13306-3.

## Background

Egypt, Africa’s third most populous country, is burdened by severe economic and healthcare challenges caused by its overpopulation [1]. The Strengthening Egypt Family Planning Program (SEFPP) is a USAID-funded initiative, which is implemented as a strategic response to the country’s rapid population growth [2, 3]. SEFPP prioritizes regions with lower family planning (FP) usage rates, such as the governorates of Upper Egypt, including Assiut. The program aims to enhance access to family planning services by expanding the availability of intrauterine devices (IUDs). A key component is training healthcare providers (HCPs) in immediate postpartum IUD (IPPIUD) insertion and establishing this practice as a routine national service during cesarean sections (CS) [4]. SEFPP places a strong emphasis on IPPIUD counseling at antenatal care (ANC), labor, and postnatal care to encourage this practice [3]. Given the rising trend of CS in Egypt, with a rate of 72% according to the Egyptian Family Health Survey (EFHS) of 2021, SEFPP leverages this opportunity for pain-free IUD insertion intraoperatively while women are under anesthesia [2, 3, 5]. The IPPIUD insertion can be done within 10 min after the delivery of the placenta or within the first 48 h of postpartum period [6].

IPPIUDs offer a safe, effective, and convenient contraceptive option for postpartum women seeking to prevent unplanned pregnancies especially those who face access-related challenges to postnatal care [7, 8]. However, it is essential to recognize the drawbacks of immediate IUD insertion, including a higher expulsion rate estimated between 5% and 17% compared to 2% for delayed insertion, an increased risk of prolonged vaginal bleeding, and potential IUD side effects such as abdominal pain, irregular bleeding, and cramping [9]. The postpartum period presents a unique opportunity for IUD insertion as women are often highly motivated to prevent subsequent pregnancies and are already in healthcare facilities with skilled providers [10]. Delaying contraception increases the risk of unintended pregnancy, considering the rapid return of fertility in non-breastfeeding women [11]. According to the EFHS-2021, the unmet need for FP is 14%, with a higher rate of 17% reported in Upper Egypt [5].

Similar programs have been implemented in many low- and middle-income countries (LMICs) such as Kenya, Sri Lanka, Nepal, Tanzania, Bangladesh, India, Rwanda, Ethiopia and El Salvador [12–15]. Unfortunately, many studies have shown that despite the efforts to promote the use of IPPIUD, usage rates vary significantly with some regions experiencing low uptake compared to others [8, 16, 17]. The percentage of IPPIUD use was 35%, 31.6%, 4%, 3.4%, 1.1%, and 0.3% in Ethiopia, Tanzania, Rwanda, Zambia, Kenya, and Eritria respectively [18–21]. In a study done in Assiut in 2020, 54.5% of postpartum women utilized PPFP methods during the first year after delivery, about 45% of users-initiated FP usage within the first six postpartum weeks and only 38.3% of those women chose the IUD [22].

The uptake of IPPIUD may be low due to several barriers including sociodemographic factors and cultural norms such as husband’s opposition or other family members, religious beliefs and lack of knowledge about FP, and reproductive barriers such as preferring high fertility and early pregnancy, in addition to method-related concerns like fear of side effects and the need for follow-up checkups [23, 24]. Additionally, the healthcare facility barriers include providers’ attitude, lack of training, lack of FP counseling in ANC visits, poor service quality, negative attitudes from HCPs, previous bad experiences, and lack of privacy [25, 26].

In Egypt, the adoption of IPPIUD is limited due to various factors, including societal, cultural, and religious norms that stigmatize FP [27, 28]. This may encompass beliefs such as viewing contraceptive use as morally inappropriate, the perceived ease of early childbearing, the preference for female HCPs, limited knowledge about ideal family size, and child gender-based preferences [29, 30]. The study aims to identify barriers and predictors of IPPIUD use among postpartum women in Assiut public hospitals and seeks to enhance the uptake of IPPIUD and strengthen the SEFPP, contributing to increased contraceptive prevalence in Egypt and other low- and middle-income countries (LMICs) with similar contexts.

## Methods

### Study design and setting

The study was designed as a case-control study conducted in three public hospitals located in Assiut City: Assiut University Hospital, Assiut General Hospital, and El-Eman General Hospital. The primary outcome is IUD insertion after CS among women in Assiut public hospitals. The exposures include sociodemographic factors (age, education, employment, socioeconomic status), obstetric and reproductive factors (number of pregnancies, prior family planning use, counseling received), cognitive and cultural factors, healthcare provider attitudes, and method-related concerns.

### Study population

The study population consisted of two groups: Cases, which included women who underwent CS and had IPPIUD inserted between April 2023 and August 2023, this included both copper and hormonal IUD which are offered free of charge within the SEFPP at the aforementioned hospitals, and the control group, which comprised women who also gave birth via CS at the same sites during the same period but did not use IPPIUD. Exclusion criteria included women who experienced complications post-delivery, such as postpartum hemorrhage, septicemia, or amniotic fluid embolism, as well as those with fibroids or abnormal uterine cavities. It is important to note that tubal ligation is not part of the contraceptive method mix supported by the SEFPP and is not included among the postpartum contraceptive options provided in public hospitals due to policies restricting non-essential surgery. Additionally, its acceptability is very low; according to the latest EFHS, the usage rate of tubal ligation is only about 1%, largely due to prevailing cultural factors [5].

### Sampling size and technique

The sample size was calculated based on the most common barrier to IPPIUD use identified in a study conducted at Meru Hospital, which found that the lack of knowledge about the IPPIUD accounted for 57% of non-use [31]. Using G power software version 3.1.9.4, the t-test for comparing the difference between two independent means, hypothesized effect size 0.5 (difference between mean knowledge score between women who used IPPIUD and women who didn’t use it), alpha error prob 0.05, power (1- beta error prob) 0.90, and allocation ratio 1:1. The required sample size was 140 women (70 women are cases and 70 are controls) in each of the hospitals.

A census list from the three studied sites, including all women who gave birth via CS from April 2023 to August 2023 was obtained, and a sample of 420 clients, 140 in each of the three hospitals, was collected via a systematic random sampling technique. Only women who consented to participate in the study and completed the questionnaire were included. Women were contacted until the required sample size was fulfilled. A pilot study was performed on 5% of the sample size to test the feasibility of the questionnaire. The results of the pilot study were not included in the analysis. Data collection occurred through telephone interviews, utilizing a semi-structured questionnaire that was administered to both cases and controls.

### The questionnaire

The questionnaire was semi-structured and consisted of three distinct sections designed to gather data relevant to the research objectives comprehensively. It was specifically developed for this study, adapted from existing literature. As detailed in Supplementary File 1, The first section focused on sociodemographic data including the women’s ages, their educational levels, employment statuses, and residential locations. Additionally, it encompassed the age, education, and occupation of their husbands, as well as the type of family structure and socioeconomic status (SES), assessed using the Family Affluence Scale III (FAS III). FAS III comprises six questions, with responses aggregated to create an FAS index that ranges from 0 to 13. A higher score on this index indicates a higher SES. The mean FAS index was employed to compare SES across different groups, and the reliability of the scale was confirmed with a Cronbach’s alpha of 0.74 [32].

The second section of the questionnaire addressed obstetric and contraceptive history, capturing critical information such as the number of pregnancies, the number of living children, and any history of abortions or unintended pregnancies. It also explored the respondents’ desires for future children, the husband opinion regarding FP use, the site of ANC, prior use of any FP methods, particularly IUD, and the counseling received regarding the IPPIUD and its timing [31, 33].

The third section identified the barriers that hinder the uptake of IPPIUD. These barriers were categorized into three main groups: cognitive and cultural barriers, HCPs and facility-related barriers, and method-related barriers. To gain a comprehensive understanding of these obstacles, the same questions were asked to both non-users and users of the IPPIUD, this approach aimed to identify the actual barriers faced by women who had not chosen to use the IPPIUD, as well as the perceived barriers that users believe other women in the community encounter. Cognitive and cultural barriers included knowledge about FP and IPPIUD, sources of information, counseling experiences, fertility preferences, familial disapproval, and prevalent myths regarding FP [31, 34]. Provider and facility barriers encompass issues such as the personal preferences of HCPs, negative attitudes from hospital staff, perceived quality of care, and privacy concerns during examinations [35]. Lastly, method-related barriers involved fears related to side effects, skepticism regarding the efficacy of IUDs, and misconceptions about the need for follow-up care [36].

### Data management and statistical analysis

Data entry, cleaning, revising, recording of variables, and statistical analysis were performed using IBM SPSS (Statistical Package for the Social Sciences) software, version 26 for Windows. Qualitative data were expressed as frequencies and percentages. Numerical data was tested for normality using Shapiro-Wilk tests and expressed by mean ± SD or median and range according to their distribution. Chi-square (χ2) and Fisher’s Exact tests were used to compare qualitative variables. T-tests and Mann Whitney-tests were used to compare mean/median difference between two groups. Univariate Logistic regression analysis was performed to identify possible factors associated with the use of IPPIUD, and significant variables entered in a multivariate backward LR logistic regression analysis to identify significant predictors of IPPIUD utilization among postpartum women. The dependent variable was defined as whether a woman utilized the IPPIUD (yes/no), while independent variables included sociodemographic factors, obstetric history, and knowledge about family planning. To assess the predictive power and fit of our model, we calculated relevant metrics such as the Akaike Information Criterion (AIC) and Bayesian Information Criterion (BIC) to assess model performance and appropriateness. The odds ratios (OR) and adjusted odds ratios (AOR) were reported along with their 95% confidence intervals (CI) to indicate the strength of associations between predictors and IPPIUD utilization. The level of significance was considered at P value < 0.05.

## Results

A total of 420 women participated in the study, with an equal distribution of 50% cases and 50% controls, comprising 140 participants from each hospital.

### Sociodemographic characteristics of the studied groups

The analysis revealed notable differences in sociodemographic characteristics between users and non-users of the IPPIUD as Table 1 shows that there is a statistically significant higher percentage of IPPIUD usage among women who had higher mean age compared to non-users (34.00 ± 5.81 vs. 28.14 ± 5.23, respectively; *P*-value < 0.001), had a secondary or higher level of education (81.4% vs. 35.2%, respectively; *P*-value < 0.001), lived in urban areas (76.7% vs. 32.9%, respectively; *P*-value < 0.001), employed (52.9% vs. 20.5%, respectively; *P*-value < 0.001), had husbands with a higher mean age (37.94 ± 5.78 vs. 34.62 ± 5.24, respectively; *P*-value < 0.001), had husbands with a secondary or higher education (87.1% vs. 45.2%, respectively; *P*-value < 0.001) and husbands who were present at home daily or weekly, lived in a nuclear family, and had higher SES compared to non-users. As for comorbidities such as diabetes, hypertension and autoimmune diseases, there was no significant difference between cases and controls.

**Table 1:** Association between the sociodemographic characteristics and IPPIUD usage among postpartum women in Assiut city, Egypt

Variable	IPPIUD users (*n* = 210)	IPPIUD non-users (*n* = 210)	*P*-Value*
Mother age in years	34.00 ± 5.81	28.14 ± 5.23	< 0.001
■ < 30	59 (28.1%)	134 (63.8%)	< 0.001
■ ≥ 30	151 (71.9%)	76 (36.2%)	
Women Education			
■ Below secondary education	39 (18.6%)	136 (64.8%)	< 0.001
■ Secondary and above	171 (81.4%)	74 (35.2%)	
Residence			
■ Urban	161 (76.7%)	69 (32.9%)	< 0.001
■ Rural	49 (23.3%)	141 (67.1%)	
Employment			
■ employed	111 (52.9%)	43 (20.5%)	< 0.001
■ Not employed	99 (47.1%)	167 (79.5%)	
Husband age in years	37.94 _±_ 5.780	34.62 ± 5.246	< 0.001
Husband education			
■ Below secondary	27 (12.9%)	115 (54.8%)	< 0.001
■ Secondary and above	183 (87.1%)	95 (45.2%)	
Husband presence at home			
■ Daily/weekly	210 (100.0%)	157 (74.8%)	< 0.001
■ Monthly or more	0 (0.0%)	53 (25.2%)	
Type of family			
■ Nuclear family	193 (91.9%)	148 (70.5%)	< 0.001
■ Extended family	17 (8.1%)	62 (29.5%)	
Presence of morbidities	36 (17.1%)	27 (12.9%)	0.219
Socioeconomic level (FAS III)	4.00 (1–12)	2.00 (0–9)	< 0.001

*Chi square test, Student T- test and Man Whitney U test. Statistical significance (*p* < 0.05).

### Obstetric and reproductive history of the studied groups

The reproductive health characteristics varied among the studied groups, Table 2 shows that there was a statistically significant higher percentage of IPPIUD usage among women who had three or more deliveries compared to non-users (85.2% vs. 61.4%, respectively; *P*-value < 0.001), had three or more living children (79.5% vs. 47.6%, respectively; *P*-value < 0.001), having at least one male child (95.7% vs. 61.4%, respectively; *P*-value < 0.001), had no history of abortions (77.6% vs. 36.2%, respectively; *P*-value < 0.001), had history of unintended pregnancies before (80.5% vs. 19.0%, respectively; *P*-value < 0.001), used FP methods before compared to non-users (76.7% vs. 24.3%, respectively; *P*-value < 0.001), among women who were using a contraceptive method prior to this delivery (65.7% vs. 20.0%, respectively; *P*-value < 0.001), and women whose reason to stop their last contraceptive method, used before this pregnancy, was either becoming pregnant while using it or experiencing side effects. Also, there was a statistically significant higher percentage of IPPIUD usage among women who did not want another baby or preferred to wait at least two years before having their next child compared to non-users.

**Table 2:** Association between the reproductive history and IPPIUD usage among postpartum women in Assiut city, Egypt

Variable	IPPIUD users (*n* = 210)	IPPIUD non-users (*n* = 210)	*P*-Value*
Parity			
■ < 3	31 (14.8%)	81 (38.6%)	< 0.001
■ ≥ 3	179 (85.2%)	129 (61.4%)	
Total number of living Children			
■ < 3	43 (20.5%)	110 (52.4%)	< 0.001
■ ≥ 3	167 (79.5%)	100 (47.6%)	
Number of male children:			
■ Do not have boys	9 (4.3%)	81 (38.6%)	< 0.001
■ Have ≥ one male child	201 (95.7%)	129 (61.4%)	
History abortions	76 (36.2%)	163 (77.6%)	< 0.001
History of unintended pregnancy before	169 (80.5%)	40 (19.0%)	< 0.001
Ever used FP before	161 (76.7%)	51 (24.3%)	< 0.001
Ever used IUD before *n* = 212	72 (44.7%)	16 (21.4%)	0.092
IUD caused side effects *n* = 88	0 (0.0%)	14 (87.5	< 0.001
Used FP method before this pregnancy	138 (65.7%)	42 (20.0%)	< 0.001
Why she stopped using FP method *n* = 180			< 0.001
■ Desire to get pregnant	54 (39.1%)	26 (61.9%)	
■ Did not stop (pregnancy on top of method)	63 (45.7%)	13 (31.0%)	
■ Side effects	21 (15.2%)	3 (7.1%)	
Does she want another baby			< 0.001
■ Yes	59 (28.1%)	152 (72.4%)	
■ No	113 (53.8%)	26 (12.4%)	
■ Not decided	38 (18.1%)	32 (15.2%)	

*Chi square/Fisher Exact test. Statistical significance (*p *< 0.05).

### FP knowledge and counseling among the studied groups

The association between the knowledge about FP and IPPIUD usage was studied, Table 3 shows that there was statistically significant higher percentage of usage among women who had heard or read about FP before compared to non-users (100.0% vs. 93.3%, respectively; *P*-value < 0.001), women whose source of knowledge about FP was ANC, the hospital during labor, post-natal visits and TV and social media, women whose husbands were supporters of the use of FP (90.0% vs. 33.3%, respectively; *P*-value < 0.001), women who attended ANC at the same hospital where they delivered (85.7% vs. 75.7%, respectively; *P*-value 0.008), women who had counseling on IPPIUD (100.0% vs. 33.3%, respectively; *P*-value < 0.001), and women who had their IPPIUD counseling during ANC (84.3% vs. 17.1%, respectively; *P*-value < 0.001), and during labor in the hospital (58.1% vs. 34.2%, respectively; *P*-value < 0.001) compared to non-users.

**Table 3:** Association between FP knowledge, husbands’ opinion, counseling and IPPIUD use among postpartum women in Assiut

Variable	IPPIUD user (*n* = 210)	IPPIUD non-user (*n* = 210)	*P*-Value*
Knowledge about FP	210 (100.0%)	196 (93.3%)	< 0.001
Source of knowledge # *n* = 210 *n* = 196			
■ From ANC visits	156 (74.3%)	40 (19.0%)	< 0.001
■ During labor	80 (38.1%)	21 (10.0%)	< 0.001
■ Postnatal visits	41 (18.6%)	7 (3.6%)	< 0.001
■ TV and social media	133 (63.3%)	105 (51.4%)	0.041
■ friends and family	195 (92.9%)	188 (91.1%)	0.182
■ FP Campaigns	39 (47.0%)	44 (22.4%)	0.333
Husband’s opinion regarding FP use			
■ Supporter	189 (90.0%)	70 (33.3%)	< 0.001
■ Opposer	21 (10.0%)	140 (66.7%)	
Attending ANC at the same hospital	180 (85.7%)	159 (75.7%)	0.008
Received counseling about IPPIUD	210 (100.0%)	70 (33.3%)	< 0.001
Time of counseling#	*N* = 210	*N* = 70	
■ During ANC	177 (84.3%)	36 (17.1%)	< 0.001
■ During labor in hospital	122 (58.1%)	24 (34.2%)	< 0.001
■ After labor in hospital	36 (17.1%)	19 (27.1%)	0.068

*Chi square test. Statistical significance (*p* < 0.05). # multiple answers were allowed.

Among non-users, Table 4 shows that only 16.2% believed that the IUD was unsuitable for them and expressed a desire to try other methods. The most frequently mentioned alternative was oral contraceptive pills (91.1%), followed by implants.

**Table 4:** Preferred methods among Non-Users of IPPIUD among postpartum women in Assiut

	IPPIUD Non-users *N* = 210	percent
If women believe IUD isn’t suitable for them and want to try another method	34	16.2%
The methods they preferred are # *N* = 34		
■ Pills	31	91.1%
■ Implant	9	26.4%
■ Injection	1	2.9%

Data is expressed in frequency and percentage.

# means multiple answers were allowed.forediting.

### Actual vs. perceived barriers to IPPIUD utilization

Regarding the barriers of IPPIUD uptake, the non-IPPIUD users were asked about the barriers that prevented them from using IPPIUD and IPPIUD users were asked about their perception of the barriers that would stop women in their community from using the IPPIUD, they reported the following:

### Regarding cognitive and cultural related barriers

**Fig. 1:**
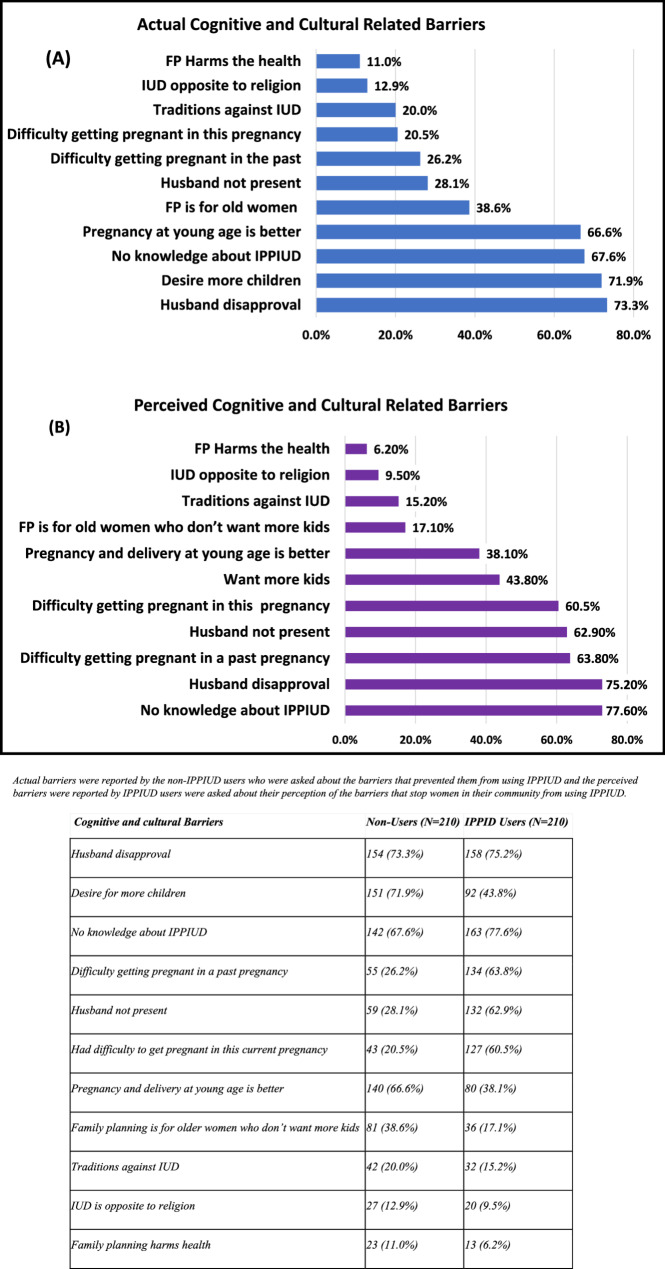
Actual vs. Perceived Cognitive and Cultural Related Barriers of IPPIUD Use

Figure 1 (A) shows that for the actual barriers, the most frequent barrier was disapproval from their husbands (73.3%). This was followed by the desire to have more children (71.9%), lack of knowledge about the IPPIUD usage (67.6%), a preference for pregnancy and delivery at a young age (66.6%), and the belief that FP is for older women who don’t want more children (38.6%). However, Fig. 1 (B) shows that the most frequently recorded perceived barrier by the IPPIUD users was the absence of knowledge about IPPIUD (77.6%), followed by disapproval from their husbands (75.2%), difficulty getting pregnant in a past pregnancy (63.8%), and the absence of the husband from home (62.9%).

Actual barriers were reported by the non-IPPIUD users who were asked about the barriers that prevented them from using IPPIUD and the perceived barriers were reported by IPPIUD users were asked about their perception of the barriers that would stop women in their community from using the IPPIUD.

### Regarding HCPs and health facilities related barriers

**Fig. 2:**
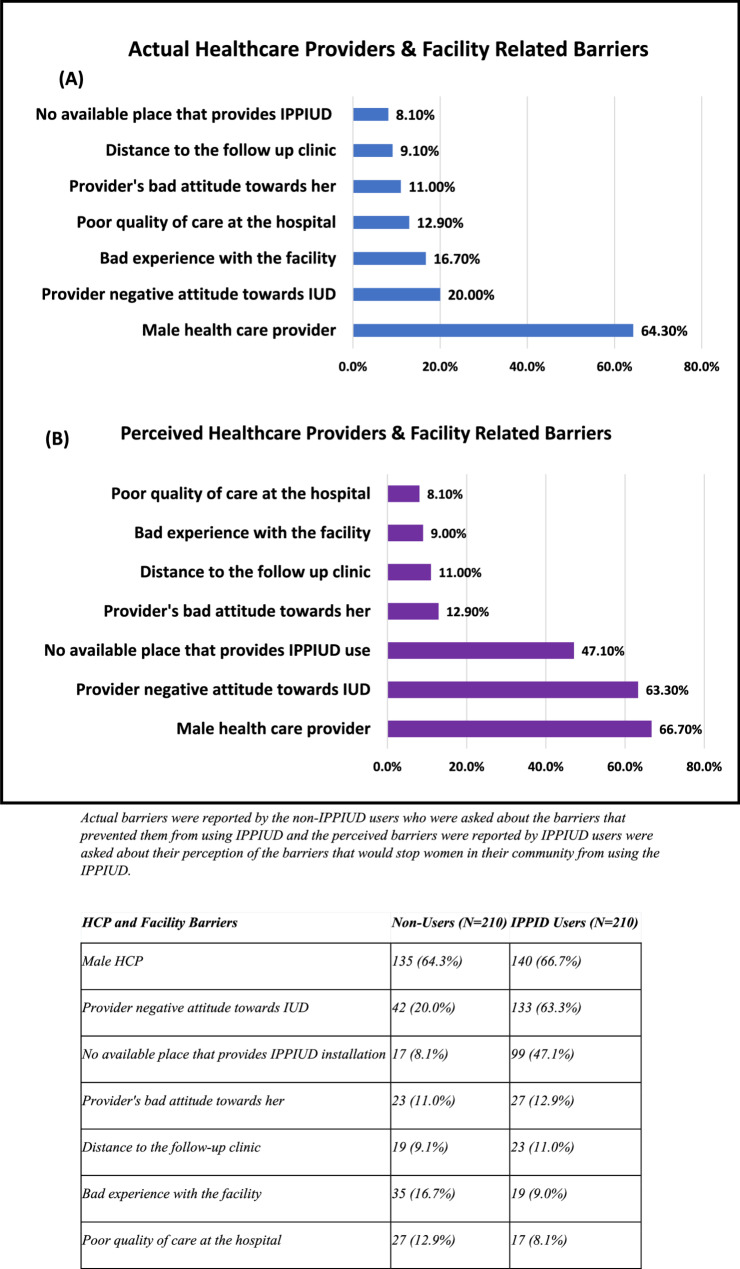
Actual vs. Perceived HCPs and Facilities Related barriers of IPPIUD Use

Figure 2 (A) shows that for the actual barriers, the primary concern was a preference against having a male HCP (64.3%), followed by the provider’s negative attitude towards IUD (20.0%) and having had a previous bad experience at the facility (16.7%). For the perceived barriers, Fig. 2 (B) shows that they were mainly due to having a male HCP (66.7%), followed by the provider’s negative attitude towards the IUD (63.3%) and lack of accessibility (47.1%).

The barriers that prevented them from using IPPIUD and the perceived barriers were reported by IPPIUD users were asked about their perception of the barriers that would stop women in their community from using the IPPIUD.

### Regarding method related barriers

**Fig. 3:**
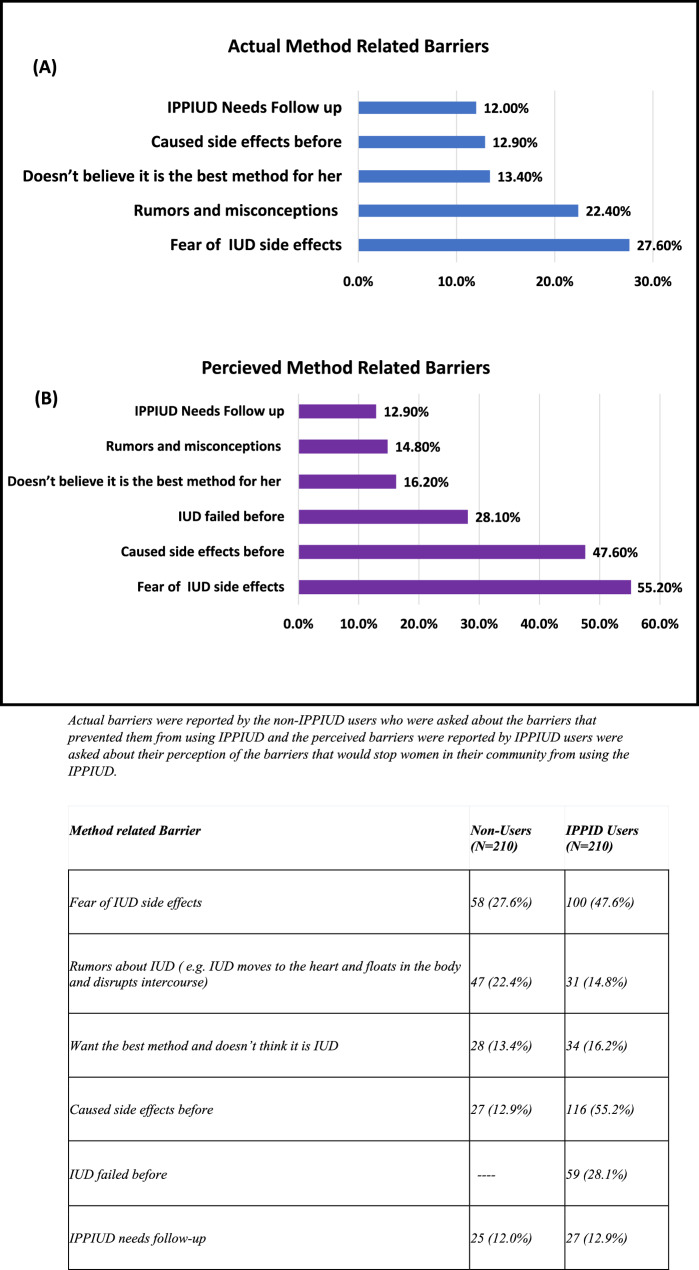
Actual vs. Perceived Method Related Barriers of IPPIUD Use

Figure3 (A) shows thatfor the actual barriers,they were mainly fear of side effects (27.6%), this was followed by rumors about the IUD (22.4%), and not believing that the IUD is the best suitable method for them (13.4%). For the perceived barriers, Fig. 3 (B) shows that they were due to fear of side effects (55.2%), followed by previous experiences of side effects from the IUD (47.6%) and that IUD could have failed before (28.1%).

Actual barriers were reported by the non-IPPIUD users who were asked about the barriers that prevented them from using IPPIUD and the perceived barriers were reported by IPPIUD users were asked about their perception of the barriers that would stop women in their community from using the IPPIUD

### Predictors of IPPIUD utilization

When the significant variables in univariate logistic regression were entered in multivariable logistic regression (Back LR) as shown in Table 5, the significant predictors of IPPIUD use among postpartum women were receiving counseling on the use of IPPIUD during ANC (OR = 11.42 (4.58–28.49; *P*-value < 0.001), having their husbands’ support for FP use (OR = 9.43 (3.44–25.83); *P*-value < 0.001), receiving IPPIUD counseling during labor (OR = 5.63 (2.56–12.33); *P*-value < 0.001), having at least one male child (OR = 4.24 (1.37–13.14); *P*-value 0.012), their source of knowledge about FP was from ANC (OR = 2.39 (1.89–5.83), *P*-value 0.034), having high SES (OR = 1.54 (1.23–1.95); *P*-value < 0.001), and increased women’s age (OR = 1.13 (1.06–1.21); *P*-value < 0.001).

**Table 5:** Predictors of IPPIUD use among postpartum women in Assiut City, Egypt

Variable	Univariate Log reg		Multivariate log reg	
	OR (95%, CI)	*P*-Value	AOR (95%, CI)	*P*-Value
Increased women’s Age	1.20 (1.16–1.26)	< 0.001	1.13 (1.06–1.21)	< 0.001
Women with secondary education and above	8.06 (5.15–12.62)	< 0.001		
Higher SES	2.28 (1.92–2.71)	< 0.001	1.54 (1.23–1.95)	< 0.001
Urban residence	6.71(4.37–10.33)	< 0.001		
Employed women	4.35 (2.83–6.70)	< 0.001		
Increased husbands’ age	1.11 (1.07–1.16)	< 0.001		
Husbands with secondary education and above	8.21 (5.04–13.35)	< 0.001		
Nuclear family	4.76 (2.67–8.48)	< 0.001		
Husbands’ support of FP use	18.00 (10.55–30.76)	< 0.001	9.43 (3.44–25.83)	< 0.001
Parity ≥ 3	3.63 (2.262–5.81)	< 0.001		
Living children ≥ 3	5.12 (3.27–8.02)	< 0.001		
Having at least one male child	14.02 (6.80–28.91)	< 0.001	4.24 (1.37–13.14)	0.012
No history of abortions	6.12 (3.98–9.40)	< 0.001		
History of unintended pregnancies	17.52 (10.79–28.45)	< 0.001		
Used FP before	10.24 (6.54–16.05)	< 0.001		
Received IPPIUD counseling during ANC	25.92 (15.47–43.46)	< 0.001	11.42 (4.58–28.49)	< 0.001
Received IPPIUD counseling during labor	4.31 (2.81–6.62)	< 0.001	5.63 (2.56–12.33)	< 0.001
Source of knowledge about FP from ANC	11.27 (7.08–17.94)	< 0.001	2.39 (1.89–5.83)	0.034
Source of knowledge about FP during labor	5.13 (3.01–8.73)	< 0.001		
Source of knowledge about FP in postnatal care	6.55 (2.86–14.99)	< 0.001		
Source of knowledge about FP from TV and social media	1.73 (1.17–2.55)	0.006		
Had ANC at the same hospital	1.93 (1.17–3.17)	0.010		

Backward LR model. Dependent variable, IPPIUD utilization.

*OR* (odds ratios), *AOR* adjusted odds ratio. 95% *CI* 95% confidence intervals.

## Discussion

The IPPIUD is an effective and safe option for FP during the postpartum period, yet it is frequently missed as a vital opportunity for mothers to take control of their reproductive health. In our study on the barriers to IPPIUD use, we identified key cognitive and cultural obstacles, including husbands’ disapproval, a desire for more children, and a lack of knowledge about IPPIUD. Among HCPs and facility-related barriers, preferences against male providers, negative attitudes towards IUDs, and previous bad experiences were the most common. Method-related barriers included fears of side effects, prevalent rumors about IUD, and doubts about the IUD’s suitability. A study conducted in Egypt revealed that concerns about potential side effects such as infection and bleeding, and fears of future infertility significantly contribute to the decline in IUD usage [27]. In Upper Egypt, many providers exhibited bias towards specific contraceptive methods, often discouraging young or nulliparous clients from using contraception until their fertility was established [37]. Similarly, research at Meru Hospital in Kenya identified barriers to IPPIUD uptake, which include provider-related issues like inadequate contraception services and counseling, method-related factors such as preferences for alternative contraceptives due to past negative experiences, and client-related challenges, including a lack of information about IPPIUD [31]. A study evaluating barriers to IPPIUD use among pregnant women in Ethiopia identified primary obstacles such as preferences for alternative methods post-childbirth, concerns about health risks, and fears of future fertility impairment [38]. Additionally, the involvement of male HCPs in IUD insertion posed a barrier for many women, as some husbands preferred male obstetricians for the CS but were reluctant to allow male physicians to perform the IUD insertion. This reluctance stemmed from a lack of understanding of the procedure, highlighting a significant gap in FP education for men [39].

Our study found that women who received counseling about IPPIUD and its benefits were more likely to use it, especially if it was during ANC visits and labor admission. These findings align with a study in Assiut, where women who received PPFP counseling during ANC and delivery were more likely to use PPFP. The study recommended integrating PPFP counseling within MCH services [22]. Another study in Ethiopia found that receiving counseling during ANC and before delivery was associated with immediate postpartum long-acting reversible contraceptive use [38]. Similarly, a study in Bahir Dar City, Northwest Ethiopia, revealed that ANC counseling on FP increased PPFP use [40]. Counseling is crucial as it informs women about the benefits of IPPIUD. Providing counseling during ANC allows women to explore contraceptive options before the stress of delivery. Moreover, during labor admission, the impending arrival of a new baby can heighten women’s sense of responsibility for their health and family well-being, influencing their motivation to consider IPPIUD. This timing enables them to make informed decisions without the pressure of post-labor recovery. One-on-one counseling in high-income settings has been shown to empower women by providing tailored information, enabling them to make informed choices about contraceptive methods like the IUD. This approach respects women’s autonomy and ensures that decisions align with their personal reproductive goals [41].

In our study, we found that spousal support was a significant predictor of IPPIUD uptake, aligning with the findings from a study in done Assiut, where husbands’ support for FP was linked to increased use of PPFP methods [22]. Research in Egypt indicates that husbands often serve as decision-makers regarding family size and sexual activity, but many lack knowledge about the benefits of FP, which can lead to their disapproval of contraceptive methods [42]. This disapproval can deter women from using IPPIUD due to concerns about marital conflict and the desire to maintain harmony.

We also found that having at least one male child was a significant predictor for IPPIUD uptake. This is consistent with findings from a study in Assiut, where women who had sons were more likely to use PPFP [22]. A qualitative study in Kenya suggested that cultural bias towards male offspring may reduce FP use, as families often continue having children until a son is born [43]. Similar findings were observed in studies done in Sudan and China [44, 45].

In our study we found that women who had knowledge about FP are more likely to use IPPIUD compared to non-users and getting her knowledge about FP from ANC visits, from HCPs, was a significant predictor for IPPIUD uptake. This aligns with a study in Egypt which revealed that women’s knowledge and understanding of FP methods and reproductive issues are critical factors influencing their use of FP methods [46]. Furthermore, a study at Meru, Kenya found that having no knowledge about IPPIUD was a significant barrier to using IPPIUD among postpartum women [38]. Notably, this knowledge deficit, reported by 67.6% of respondents, may contribute both to the low uptake of IPPIUD and to limited awareness of potential complications, including device expulsion.

We also found that women from families with high SES significantly predicts IPPIUD uptake. This aligns with studies conducted in Assiut and Dakahlia Governorates in Egypt [22, 47, 48]. Women from higher SES backgrounds typically have better access to education and healthcare, and they are more likely to be employed and possess greater autonomy and empowerment in making health and FP decisions.

Our study identified increased maternal age as a significant predictor for IPPIUD use. This finding aligns with a study carried out in several villages across the Assiut and Sohag governorates in Egypt, which showed that HCPs advised against the use of FP methods for women under the age of 20 and suggested the use of these methods after the age of 35 [37]. Moreover, another study examining the patterns of use and obstacles to FP in Egypt found that, the age group of 35–39 had the highest level of ever having used any FP method [49]. Also, we found similar results in LMICs [7, 50].

This study has several strengths; it utilizes comparative analysis that effectively controls for confounding variables, enhancing the reliability of our findings. With a robust sample size of 420 women from three public hospitals in Assiut City, it captures a diverse population, enriching data on sociodemographic factors and cultural attitudes toward family planning (FP) use. Pilot testing of the semi-structured questionnaire, and advanced statistical techniques such as logistic regression analyses contribute to the strength of our research. Also, the study has some limitations, including the absence of providers’ perspectives on barriers, the need for qualitative data to complement the findings, and a limited exploration of structural barriers and broader issues such as healthcare system policies and resource allocation. Furthermore, while the study acknowledged some aspects of partner involvement, such as husband or family disapproval, it did not thoroughly examine the perspectives of male partners regarding contraception.

## Conclusions

The main barriers to IPPIUD use include disapproval from husbands, a desire for more children, a lack of IPPIUD knowledge, preferences against male HCPs, provider negativity towards IUDs, prior bad experiences at facilities, fear of side effects, prevalent rumors, and doubts about IUD suitability. However, the predictors of increased IPPIUD uptake include receiving counseling on IPPIUD use during ANC and labor, having the husband’s support for FP use, having male children, obtaining FP knowledge from ANC, having high SES, and increased women’s age.

We recommend that policymakers should promote FP education, particularly targeting men, and address gender norms, while utilizing social media platforms for outreach. Healthcare sector should focus on training HCPs to utilize the postpartum period and labor for effective FP counseling, use FP educational videos within waiting rooms to increase counseling rates, and recruit more female providers to meet patient preferences. Further research should examine structural barriers and investigate the role of postpartum care in promoting continued IPPIUD use.

## Supplementary Information

Supplementary Material 1.

## Data Availability

The datasets used and analyzed during this study are available from the corresponding author upon reasonable request.

## Abbreviations

ANC: Antenatal care
CS: Cesarian section
EFHS: Egyptian family health survey
FAS III: Family Affluence Scale III
FP: Family planning
HCPs: Healthcare providers
IPPIUD: Immediate postpartum IUD
IUDs: Intrauterine devices
LMICs: Low- and Middle-Income Countries
PPFP: Postpartum family planning
SEFPP: Strengthening Egypt's Family Planning Program
SES: Socioeconomic status
USAIDs: U.S. Agency for International Development.

## Declarations

Before starting the questionnaire, the study and its aim were thoroughly explained to each participant, ensuring they understood the purpose of their participation. Informed verbal consent was obtained from those who agreed to participate in the study. Participants were assured of privacy and confidentiality, which were maintained by removing their names from the data entry file and using only identification numbers.

## References

[1] El-Attar EA, Helmy Elkaffas RM, Aglan SA, Naga IS, Nabil A, Abdallah HY. Genomics in egypt: current status and future aspects. Front Genet. 2022;13:797465. 10.3389/FGENE.2022.797465/BIBTEX. 35664315 10.3389/fgene.2022.797465 PMC9157251

[2] USAID/Egypt. Strengthening Egypt’s Family Planning Program. Activity Fact Sheet. Cairo: United States Agency for International Development. 2020. Available from: https://darpe.me/project-entries/strengthening-egypts-family-planning-program-sefpp/.

[3] Strengthening Egypt’s Family Planning Program (USAID). - JSI n.d. https://www.jsi.com/project/strengthening-egypts-family-planning-program-sefpp/ (accessed April 11, 2024).

[4] STRENGTHENING, EGYPT’S. FAMILY PLANNING PROGRAM (SEFPP) AY5 Annual Report 2022.

[5] Arab Republic of Egypt - Health Survey for the Egyptian Households. 2021 n.d. https://censusinfo.capmas.gov.eg/Metadata-en-v4.2/index.php/catalog/665 (accessed July 7, 2024).

[6] Abebaw Y, Berhe S, Abebe SM, Adefris M, Gebeyehu A, Gure T, et al. Providers’ knowledge on postpartum intrauterine contraceptive device (PPIUCD) service provision in Amhara region public health facility, Ethiopia. PLoS One. 2019. 10.1371/JOURNAL.PONE.0214334. 30946759 10.1371/journal.pone.0214334 PMC6449033

[7] Shiferaw Y, Jisso M, Fantahun S, Eshetu B, Assefa AA, Gebretsadik A. Acceptance, utilization, and factors associated with immediate postpartum intrauterine contraceptive device among mothers delivered at public health facilities in Hawassa city, ethiopia: Institution-based study. Reprod Health. 2023;20:1–11. 10.1186/S12978-023-01586-Z/TABLES/6. 10.1186/s12978-023-01586-z PMC9996894 36890509

[8] Gallagher MC, Morris CN, Fatima A, Daniel RW, Shire AH, Sangwa BMM. Immediate postpartum Long-Acting reversible contraception: A comparison across six humanitarian country contexts. Front Glob Womens Health. 2021;2:613338. 10.3389/FGWH.2021.613338/BIBTEX. 34816183 10.3389/fgwh.2021.613338 PMC8593990

[9] Sothornwit J, Kaewrudee S, Lumbiganon P, Pattanittum P, Averbach SH. Immediate versus delayed postpartum insertion of contraceptive implant and IUD for contraception. Cochrane Database Syst Rev. 2022;10(10):CD011913. 10.1002/14651858.CD011913.pub3. CD011913.pub3. PMID: 36302159; PMCID: PMC9612833. 36302159 10.1002/14651858.CD011913.pub3 PMC9612833

[10] ACOG Practice Bulletin No. 121: Long-acting reversible contraception: Implants and intrauterine devices. Obstetrics and Gynecology 2011;118:184–96. 10.1097/AOG.0B013E318227F05E 10.1097/AOG.0b013e318227f05e 21691183

[11] Teal S, Edelman A. Contraception selection, effectiveness, and adverse effects: a review. JAMA. 2021;326:2507–18. 10.1001/JAMA.2021.21392. 34962522 10.1001/jama.2021.21392

[12] Scaling Up Immediate Postpartum Family Planning Services in Rwanda n. d. https://mcsprogram.org/resource/scaling-up-immediate-postpartum-family-planning-services-in-rwanda/ (accessed July 7, 2024).

[13] Eva G, Gold J, Makins A, Bright S, Dean K, Tunnacliffe EA, Fatima P, Yesmin A, Muganyizi P, Kimario GF, Dalziel K. Economic Evaluation of Provision of Postpartum Intrauterine Device Services in Bangladesh and Tanzania. Glob Health Sci Pract. 2021;9(1):107–22. 10.9745/GHSP-D-20-00447. PMID: 33795364; PMCID: PMC8087427. 33795364 10.9745/GHSP-D-20-00447 PMC8087427

[14] Ethiopia Basic Emergency Obstetric. and Newborn Care EOP Summary & Results 2016.

[15] de Caestecker L, Banks L, Bell E, Sethi M, Arulkumaran S. Planning and implementation of a FIGO postpartum intrauterine device initiative in six countries. Int J Gynaecol Obstet. 2018;143:4–12. 10.1002/IJGO.12598. 30225869 10.1002/ijgo.12598

[16] Guye AH, Kanea EB, Nigussie T, Girma D, Shambi DB. Utilization of immediate postpartum intrauterine device and its associated factors among women who gave birth in public hospitals in West Wollega Zone, Oromia, Ethiopia. Front Med (Lausanne). 2023;10:1238496. 10.3389/fmed.2023.1238496. PMID: 38076264; PMCID: PMC10701423. 38076264 10.3389/fmed.2023.1238496 PMC10701423

[17] Huber-Krum S, Hackett K, Senderowicz L, Pearson E, Francis JM, Siril H, et al. Women’s perspectives on postpartum intrauterine devices in Tanzania. Stud Fam Plann. 2019;50:317. 10.1111/SIFP.12106. 31755132 10.1111/sifp.12106 PMC6972629

[18] Ethiopia Mini Demographic and Health Survey (2019 EMDHS) n.d.

[19] Pearson E, Senderowicz L, Pradhan E, Francis J, Muganyizi P, Shah I, et al. Effect of a postpartum family planning intervention on postpartum intrauterine device counseling and choice: evidence from a cluster-randomized trial in Tanzania. BMC Womens Health. 2020. 10.1186/S12905-020-00956-0. 32398077 10.1186/s12905-020-00956-0 PMC7218519

[20] Tolefac PN, Nana TN, Yeika EV, Awungafac NS, Ntsama Y, Njotang PN. Trends and patterns of family planning methods used among women attending family planning clinic in a rural setting in sub-Sahara Africa: the case of Mbalmayo district hospital, Cameroon. BMC Res Notes. 2018;11:541. 10.1186/S13104-018-3658-1. 30068386 10.1186/s13104-018-3658-1 PMC6071381

[21] Costa V, Da, Ingabire R, Sinabamenye R, Karita E, Umutoni V, Hoagland A, et al. An exploratory analysis of factors associated with interest in postpartum intrauterine device uptake among pregnant women and couples in kigali, Rwanda. Clin Med Insights Reprod Health. 2019;13:117955811988684. 10.1177/1179558119886843. 10.1177/1179558119886843 PMC6893932 31839717

[22] Mohammed HM, Zaky MA, Hany AM. Postpartum family planning among women attending maternal and child health centers in Assiut governorate, upper Egypt. J Egypt Public Health Assoc. 2024;99:13. 10.1186/s42506-024-00160-0. 38853225 10.1186/s42506-024-00160-0 PMC11162981

[23] Eluwa GIe, Atamewalen R, Odogwu K, Ahonsi B. Success Providing Postpartum Intrauterine Devices in Private-Sector Health Care Facilities in Nigeria: Factors Associated With Uptake. Glob Health Sci Pract. 2016;4(2):276–83. 10.9745/GHSP-D-16-00072. PMID: 27353620; PMCID: PMC4982251. 27353620 10.9745/GHSP-D-16-00072 PMC4982251

[24] Divakar H, Bhardwaj A, Purandare CN, Sequeira T, Sanghvi P. Critical factors influencing the acceptability of post-placental insertion of intrauterine contraceptive device: a study in six public/private institutes in India. J Obstet Gynaecol India. 2019;69:344–9. 10.1007/S13224-019-01221-7. 31391742 10.1007/s13224-019-01221-7 PMC6661034

[25] Chitashvili T, Holschneider S, Clark PA. Improving quality of postpartum family planning in low-resource settings: a framework for policy makers, managers, and medical care providers. Bethesda (MD): University Research Co., LLC (URC), USAID ASSIST Project. 2016:19.

[26] Cooper M, Cameron S. Successful implementation of immediate postpartum intrauterine contraception services in Edinburgh and framework for wider dissemination. Int J Gynaecol Obstet. 2018;143:56–61. 10.1002/ijgo.12606. 30225868 10.1002/ijgo.12606

[27] Elkhateeb RR, Kishk E, Sanad A, Bahaa H, Hagazy AR, Shaheen K, et al. The acceptability of using IUDs among Egyptian nulliparous women: A cross-sectional study. BMC Womens Health. 2020;20:1–6. 10.1186/S12905-020-00977-9/TABLES/4. 32503576 10.1186/s12905-020-00977-9 PMC7275565

[28] Accelerating Access to Postpartum Family Planning Meeting Report - Family Planning. 2030 n.d. https://www.fp2030.org/resources/accelerating-access-to-postpartum-family-planning/ (accessed March 28, 2024).

[29] Tafa L, Worku Y. Family planning utilization and associated factors among postpartum women in Addis Ababa, Ethiopia, 2018. PLoS One. 2021. 10.1371/JOURNAL.PONE.0245123. 33481796 10.1371/journal.pone.0245123 PMC7822255

[30] Abdel-Salam DM, Albahlol IA, Almusayyab RB, Alruwaili NF, Aljared MY, Alruwaili MS, Alnasser RM. Prevalence, Correlates, and Barriers of Contraceptive Use among Women Attending Primary Health Centers in Aljouf Region, Saudi Arabia. Int J Environ Res Public Health. 2020;17(10):3552. 10.3390/ijerph17103552. PMID: 32438740; PMCID: PMC7277515. 32438740 10.3390/ijerph17103552 PMC7277515

[31] Kirigia C, Gitonga L, Muraya MM. Barriers to immediate post-partum intra-uterine contraceptive device uptake among mothers delivering at Meru hospital. Open J Obstet Gynecol. 2019;09:312–25. 10.4236/ojog.2019.93032.

[32] Hounkponou F, Ahanhanzo YG, Biaou COA, Dos-Santos CR, Ahouingnan A, Obossou AA, et al. Postpartum contraceptive use in Parakou (A City in Northern Benin) in 2018: A community based Cross-Sectional study. Open Access J Contracept. 2019;10:19. 10.2147/OAJC.S219709. 31572028 10.2147/OAJC.S219709 PMC6756835

[33] Kanakuze CA, Kaye DK, Musabirema P, Nkubito P, Mbalinda SN. Factors associated with the uptake of immediate postpartum intrauterine contraceptive devices (PPIUCD) in rwanda: a mixed methods study. BMC Pregnancy Childbirth. 2020;20:1–11. 10.1186/S12884-020-03337-5/TABLES/3. 10.1186/s12884-020-03337-5 PMC7590736 33109097

[34] Terefe G, Wakjira D, Abebe F. Immediate postpartum intrauterine contraceptive device use among pregnant women attending antenatal clinics in Jimma town public healthcare facilities, Ethiopia: intentions and barriers. SAGE Open Med. 2023. 10.1177/20503121231157212. 36896192 10.1177/20503121231157212 PMC9989451

[35] Holden EC, Lai E, Morelli SS, Alderson D, Schulkin J, Castleberry NM, et al. Ongoing barriers to immediate postpartum long-acting reversible contraception: a physician survey. Contraception and Reproductive Medicine. 2018;3:1–6. 30455978 10.1186/s40834-018-0078-5 PMC6222995

[36] Kaydor VK, Adeoye IA, Olowolafe TA, Adekunle AO. Barriers to acceptance of post-partum family planning among women in Montserrado county, Liberia. Niger Postgrad Med J. 2018;25:143–8. 10.4103/NPMJ.NPMJ_96_18. 30264764 10.4103/npmj.npmj_96_18

[37] Aziz MM, El-Gazzar AF. Provider bias and family planning in upper egypt: a simulated client approach. J Egypt Public Health Assoc. 2023;98:1–9. 10.1186/S42506-023-00144-6/FIGURES/1. 37777657 10.1186/s42506-023-00144-6 PMC10542042

[38] Arero WD, Teka WG, Hebo HJ, Woyo T, Amare B. Prevalence of long-acting reversible contraceptive methods utilization and associated factors among counseled mothers in immediate postpartum period at Jimma university medical center, Ethiopia. Contracept Reproductive Med 2022. 2022;7:1. 10.1186/S40834-022-00184-X. 10.1186/s40834-022-00184-x PMC9438260 36050794

[39] Yates M, Styles D, Janes J, DeLozier S, Furman L. Identifying barriers to post-placental intrauterine device placement: male partners’ views. Eur J Contracept Reprod Health Care. 2020;25:159–65. 10.1080/13625187.2020.1730793. 32162558 10.1080/13625187.2020.1730793

[40] Tafere TE, Afework MF, Yalew AW. Counseling on family planning during ANC service increases the likelihood of postpartum family planning use in Bahir Dar City administration, Northwest ethiopia: a prospective follow up study. Contracept Reproductive Med 2018. 2018;3:1. 10.1186/S40834-018-0081-X. 10.1186/s40834-018-0081-x PMC6307161 30607256

[41] Hurley EA, Duello A, Finocchario-Kessler S, et al. Expanding Contraception Access for Women With Opioid-Use Disorder: A Qualitative Study of Opportunities and Challenges. Am J Health Promo. 2020;34(8):909–18. 10.1177/0890117120927327. 10.1177/0890117120927327 PMC7577934 32468826

[42] Hamed ZF, El-Gazzar AF, Moftah FM. Knowledge, attitude and practice of family planning methods among husbands in a village of Assiut Governorate. Egypt J Hosp Med 2018;73.

[43] Abdi B, Okal J, Serour G, Temmerman M. Children are a blessing from God- A qualitative study exploring the socio-cultural factors influencing contraceptive use in two Muslim communities in Kenya. Reprod Health. 2020;17:1–11. 10.1186/S12978-020-0898-Z/TABLES/1. 32245521 10.1186/s12978-020-0898-z PMC7119281

[44] Tancioco V, Perry J, Sim MS, Sridhar A. Cultural influences on family planning use. Obstet Gynecol. 2016;127:125S. 10.1097/01.AOG.0000483508.98918.BA.

[45] Ying W, Yiyin Y, Xiaojiang W, En C. What determines giving birth to a son: the social transformation of how institution and culture affect women’s fertility choices. J Chin Sociol. 2017;4(1):1–22. 10.1186/S40711-017-0057-2.

[46] Aly R, Aly M. The situation of Egyptian women in childbearing and family planning and some related factors: a perspective of correspondence analysis. Univers J Public Health. 2023;11:14–26. 10.13189/ujph.2023.110103.

[47] Hassan Abo-Rahma A, Sayed Hafez A, Abd El-Monaem El-Moselhy E, Author C. Urban and rural differences regarding family planning outcomes in Assiut district, Assiut governorate, Egypt. Al-Azhar Int Med J. 2022;3:29–34. 10.21608/AIMJ.2022.109151.1718.

[48] Farrag NS, Fathy AA, AbdelWahab F. Practice of family planning among married female attendants to Shawa Family Health Unit, Dakahlia. Egypt Egyp Fam Med J. 2020;4(1):24–41.

[49] (PDF) Trend and Pattern of Use and Barriers to Family Planning in Egypt. n.d. https://www.researchgate.net/publication/270340326_Trend_and_Pattern_of_Use_and_Barriers_to_Family_Planning_in_Egypt (accessed April 12, 2024).

[50] Da Silva ICM, Everling F, Hellwig F, Ronsmans C, Benova L, Requejo J, et al. Does women’s age matter in the SDGs era: coverage of demand for family planning satisfied with modern methods and institutional delivery in 91 low- and middle-income countries. Reprod Health. 2020;17:1–9. 10.1186/S12978-020-0903-6/TABLES/2. 32306969 10.1186/s12978-020-0903-6 PMC7168879

