# Supplementary material for: Exploring barriers and facilitators to immediate postpartum intrauterine device uptake within the strengthening Egypt family planning program: a case-control study
Source: BMC Health Serv Res. 2025 Aug 14;25:1081. doi: 10.1186/s12913-025-13306-3 (PMC12351826; doi:10.1186/s12913-025-13306-3)
Supplement: Supplementary file 1 — Supplementary Material 1. [file 12913_2025_13306_MOESM1_ESM.docx]

**Questionnaire for Exploring Barriers and Facilitators to Immediate Postpartum IUD uptake within the strengthening Egypt Family Planning Program**

| **Move to** | **Answer code** | **Question** |  | |
| --- | --- | --- | --- | --- |
| **Section 1: A-Demographic data** | | | | |
|  |  | What is your name? |  |  |
|  | Age in years. | What is your age? |  |  |
|  | 1. 1-Male. 2. 2-Female. | What is the sex of the child? |  |  |
|  | Age in months. | What is date of birth of the child? |  |  |
|  | 1. 1-Urban. 2. 2-Rural. | Where do you live? |  |  |
|  | 1. 1-Illiterate. 2. 2-Primary education. 3. 3-Secondary education. 4. 4-University degree or higher. | What is the highest educational level you have completed? |  |  |
| If no, move to Q9 | 1. 1-Yes. 2. 2-No. | Are you employed? (Paid work) |  |  |
|  |  | What is your current job? |  |  |
|  | Age in years. | How old is your husband? |  |  |
|  | 1-Illiterate.  2-Primary education.  3-Secondary education.  4-University degree or higher. | What is your husband's highest educational level? |  |  |
| If no, move to Q13 | 1. 1-Yes.   2-No. | Is your husband employed? |  |  |
|  |  | What is your husband's job? |  |  |
|  | 1-present daily or once or twice weekly.  2-present once or twice a month or annually. | Is your husband present at home regularly? |  |  |
|  | 1. 1-Nuclear family. 2. 2-Extended family. | Do you live in an apartment alone with your husband and children? (type of family) |  |  |
|  | 1. 1-Yes. 2. 2-No. | Do you have any health problems or chronic diseases for which you receive treatment? |  |  |
| **Section 1: B- Socioeconomic data** | | | | |
|  | No.................................................0 Yes................................................1 | Do you have a separate bedroom? |  | |
|  | None...............................................0 Yes, one.........................................1 Two................................................2 More than two................................3 | How many bathrooms do you have at home? |  | |
|  | No..................................................0 Yes, one.........................................1 Two or more..................................2 | Does your family own a private car, taxi, or transport vehicle? |  | |
|  | None...............................................0 Yes, one.........................................1 Two................................................2 More than two................................3 | How many computers (laptops and tablets) do you have at home? |  | |
|  | No..................................................0 Yes.................................................1 | Do you have a dishwasher? |  | |
|  | I do not travel.................................0 Once...............................................1 Twice.............................................2 More than twice.............................3 | In the past year, how many times have you traveled on vacation outside your city? |  | |
| **Section 2: Reproductive and obstetric data** | | | | |
|  |  | What is the number of pregnancies you had? |  | |
|  |  | What is the number of living children currently in the family? |  | |
|  |  | What is the number of livings sons you have? |  | |
|  |  | What is the number of abortions you had? |  | |
|  | 1-Yes.  2-No. | Have you experienced an unintended pregnancy before? |  | |
|  | 1-Yes.  2-No. | Have you heard or read about family planning methods before? |  | |
|  | 1-From ANC visits.  2-During labor.  3-Postnatal visits.  4-TV and social media.  5-Friends and family.  6-FP Campaigns. | What is the source from which you heard\learned about family planning methods? (Multiple answers allowed) |  | |
| If no, move to Q34 | 1-Yes.  2-No. | Have you used any family planning method before? |  | |
| If no, move to Q32 | 1-Yes.  2-No. | Have you used an IUD before? |  | |
|  | 1-Yes.  2-No. | Did you experience any problems or side effects from the IUD? |  | |
| If no, move to Q34 | 1-Yes.  2-No. | Did you use any contraceptive method before becoming pregnant with this child? |  | |
|  | 1-Desire to become pregnant.  2-Did not stop (became pregnant while using the method).  3-Side effects occurred. | What was the reason for stopping the method? |  | |
|  | 1-Yes.  2-No.  3-Not decided. | Do you want to have another child after the last born? |  | |
|  | 1-Supporter.  2-Opposer. | What is your husband's opinion on using family planning methods? |  | |
|  | 1-Yes.  2-No. | Did you attend antenatal care at the same hospital where you gave birth? |  | |
| If no move to Q36 | 1-Yes.  2-No. | Did you receive counselling about IPPIUD? |  | |
|  | 1-During antenatal care.  2-During labor in the hospital.  3-After labor in hospital. | What was the timing of the IPPIUD counselling? (Multiple answers allowed) |  | |
| **Section 3: Barriers that impede IPPIUD uptake** | | | | |
| For non-users: which of these reasons, that I will tell you now, contributed to your decision not to use the IPPIUD?  For users: I will read you a series of statements, and you tell me whether women in your community would refuse to use the immediate postpartum IUD for these reasons. | | | | |
| **3.A-Cognitive and cultural barriers** | | | | |
| 1-Yes.  2-No. | | Lack of awareness or knowledge about the possibility of having an IUD inserted immediately after childbirth. |  | |
| 1-Yes.  2-No. | | Due to customs and traditions followed in her country. |  | |
| 1-Yes.  2-No. | | The community prefers that women have children at a younger age. |  | |
| 1-Yes.  2-No. | | Because you\they believe that using contraception is against the religion. |  | |
| 1-Yes.  2-No. | | Due to rejection from your\her husband. |  | |
| 1-Yes.  2-No. | | Due to the husband's absence for any reason such as travel/divorce. |  | |
| 1-Yes.  2-No. | | Wanting more children because she does not have enough according to her ideal number. |  | |
| 1-Yes.  2-No. | | You\they believe that contraceptive methods harm health. |  | |
| 1-Yes.  2-No. | | It is believed that contraceptive methods are for older women who do not want more children. |  | |
| 1-Yes.  2-No. | | Faced difficulties in becoming pregnant before this pregnancy. |  | |
| 1-Yes.  2-No. | | Faced delays (long duration) and difficulties in conceiving in previous pregnancies in the past due to reproductive or fertility challenges. |  | |
| **3.B-Healthcare providers and health facility related barriers** | | | | |
| 1-Yes.  2-No. | | That only a male doctor was available to insert it for her / That women won’t approve that a male doctor would install the IUD? |  | |
| 1-Yes.  2-No. | | Had a bad experience with the healthcare provider that could negatively affect your\their decision to use the IUD? |  | |
| 1-Yes.  2-No. | | Due to negative attitudes towards the IUD from doctors or nurses at the hospital. |  | |
| 1-Yes.  2-No. | | Lack of accessibility or not knowing any healthcare facility that provides immediate postpartum IUD insertion services. |  | |
| 1-Yes.  2-No. | | Due to a previous bad experience for herself or a relative with the facility |  | |
| 1-Yes.  2-No. | | Poor quality of service at the healthcare facility providing IUD insertion services. |  | |
| 1-Yes.  2-No. | | The distance to the follow-up clinic is far from your\their home. |  | |
| **3.C-Method related barriers** | | | | |
| 1-Yes.  2-No. | | Fear of the IUD side effects. |  | |
| 1-Yes.  2-No. | | Fear due to rumors or misconceptions about the IUD such as the IUD moving to the heart. |  | |
| 1-Yes.  2-No. | | Wanting the best method and not convinced that the IUD is the best suitable method for her. |  | |
| 1-Yes.  2-No. | | She used the IUD before, and it caused her bleeding, pain, or any side effects. |  | |
| 1-Yes.  2-No. | | The IUD requires follow-up. |  | |
| 1-Yes.  2-No. | | The IUD failed before (she became pregnant). |  | |
